# Exploring the success of Brazilian endemic clone *Pseudomonas aeruginosa* ST277 and its association with the CRISPR-Cas system type I-C

**DOI:** 10.1186/s12864-020-6650-9

**Published:** 2020-03-23

**Authors:** Melise Chaves Silveira, Cláudio Marcos Rocha-de-Souza, Rodolpho Mattos Albano, Ivson Cassiano de Oliveira Santos, Ana Paula D’Alincourt Carvalho-Assef

**Affiliations:** 1 Laboratório de Pesquisa em Infecção Hospitalar, Oswaldo Cruz Institute, Fiocruz, Avenida Brasil 4365, Manguinhos, Rio de Janeiro, Rio de Janeiro 21040-360 Brazil; 2 Departamento de Bioquímica, Universidade do Estado do Rio de Janeiro, Boulevard Vinte e Oito de Setembro, 87, fundos, andar 4, Vila Isabel, Rio de Janeiro, Rio de Janeiro 20551-030 Brazil

**Keywords:** *Pseudomonas aeruginosa*, ST277, Comparative genomics, CRISPR

## Abstract

**Background:**

The Brazilian endemic clone *Pseudomonas aeruginosa* ST277 carries important antibiotic resistance determinants, highlighting the gene coding for SPM-1 carbapenemase. However, the resistance and persistence of this clone is apparently restricted to the Brazilian territory. To understand the differences between Brazilian strains from those isolated in other countries, we performed a phylogenetic analysis of 47 *P. aeruginosa* ST277 genomes as well as analyzed the virulence and resistance gene profiles. Furthermore, we evaluated the distribution of genomic islands and assessed in detail the characteristics of the CRISPR-Cas immunity system in these isolates.

**Results:**

The Brazilian genomes presented a typical set of resistance and virulence determinants, genomic islands and a high frequency of the CRISPR-Cas system type I-C. Even though the ST277 genomes are closely related, the phylogenetic analysis showed that the Brazilian strains share a great number of exclusively SNPs when compared to other ST277 genomes. We also observed a standard CRISPR spacers content for *P. aeruginosa* ST277, confirming a strong link between sequence type and spacer acquisition. Most CRISPR spacer targets were phage sequences.

**Conclusions:**

Based on our findings, *P. aeruginosa* ST277 strains circulating in Brazil characteristically acquired *In*163 and PAGI-25, which can distinguish them from strains that do not accumulate resistance mechanisms and can be found on the Asian, European and North American continents. The distinctive genetic elements accumulated in Brazilian samples can contribute to the resistance, pathogenicity and transmission success that characterize the ST277 in this country.

## Background

*Pseudomonas aeruginosa* is an important pathogen that shows a strong potential for development of multidrug resistance and is frequently implicated in healthcare-associated infections. Since the first report in 2002, SPM-1 metallo-β-lactamase is the main carbapenemase associated with *P. aeruginosa* in Brazil [1, 2]. To date, the *bla*_SPM-1_ is restricted to *P. aeruginosa* and there are only two confirmed cases outside of Brazil, both of which received medical treatment while in this country [3, 4]. Although SPM-1-producing *P. aeruginosa* has been mainly isolated from nosocomial settings, reports of this multidrug-resistant bacterium in urban rivers and microbiota of migratory birds in Brazil alert to the dispersion of this important resistance mechanism [5, 6]. Usually, the *bla*_SPM-1_ gene is inserted in the mobile genetic element ICE_Tn4371_6061 [7] and, in turn, this ICE is located in *P. aeruginosa*’s Genomic Island 15 (PAGI-15) [8].

Most SPM-1-producing *P. aeruginosa* strains descend from a common ancestor, a clone belonging to ST277 [2]. This clone has been characterized as a resistance-enriched ST [9], and the expression of SPM-1 generates resistance to all β-lactams, except for aztreonam [8]. Besides SPM-1, other genetic determinants have been associated with ST277: i) the class 1 integron *In*163 carrying three resistance genes (*aac*A4, *bla*_OXA-56_ and *aad*A7); ii) *rmtD* gene that confers high-level resistance to most aminoglycosides; and iii) the type I-C of Clustered Regularly Interspaced Short Palindromic Repeat (CRISPR) and associated proteins [9–11].

The CRISPR family of repetitive DNA sequences, together with a group of CRISPR-associated (*cas*) genes, encodes a unique defense mechanism that acts against invading genetic elements such as viruses and plasmids. CRISPR loci consist of an array of short and partially palindromic, repetitive sequences interspaced by equally short ‘spacer’ sequences from viral or plasmid origin [12–14]. The Class 1 system is widespread among Archaea and Bacteria, and includes type I, type III, as well as the rare type IV. The *cas8c* gene is the signature gene for subtype I-C which includes other six *cas* genes [15]. *P. aeruginosa* has emerged as a major CRISPR-Cas model system, with types I-F and I-E being the CRISPR-Cas system most commonly found in this species [9]*.*

Here, we examine the phylogenetic distribution and conservation of genetic determinants among ST277 *P. aeruginosa* genomes available at NCBI. We aim to provide in depth evidence about the genetic determinants that have contributed for its widespread resistance and persistence in Brazil as opposed to other countries.

## Results

**Fig. 1:**
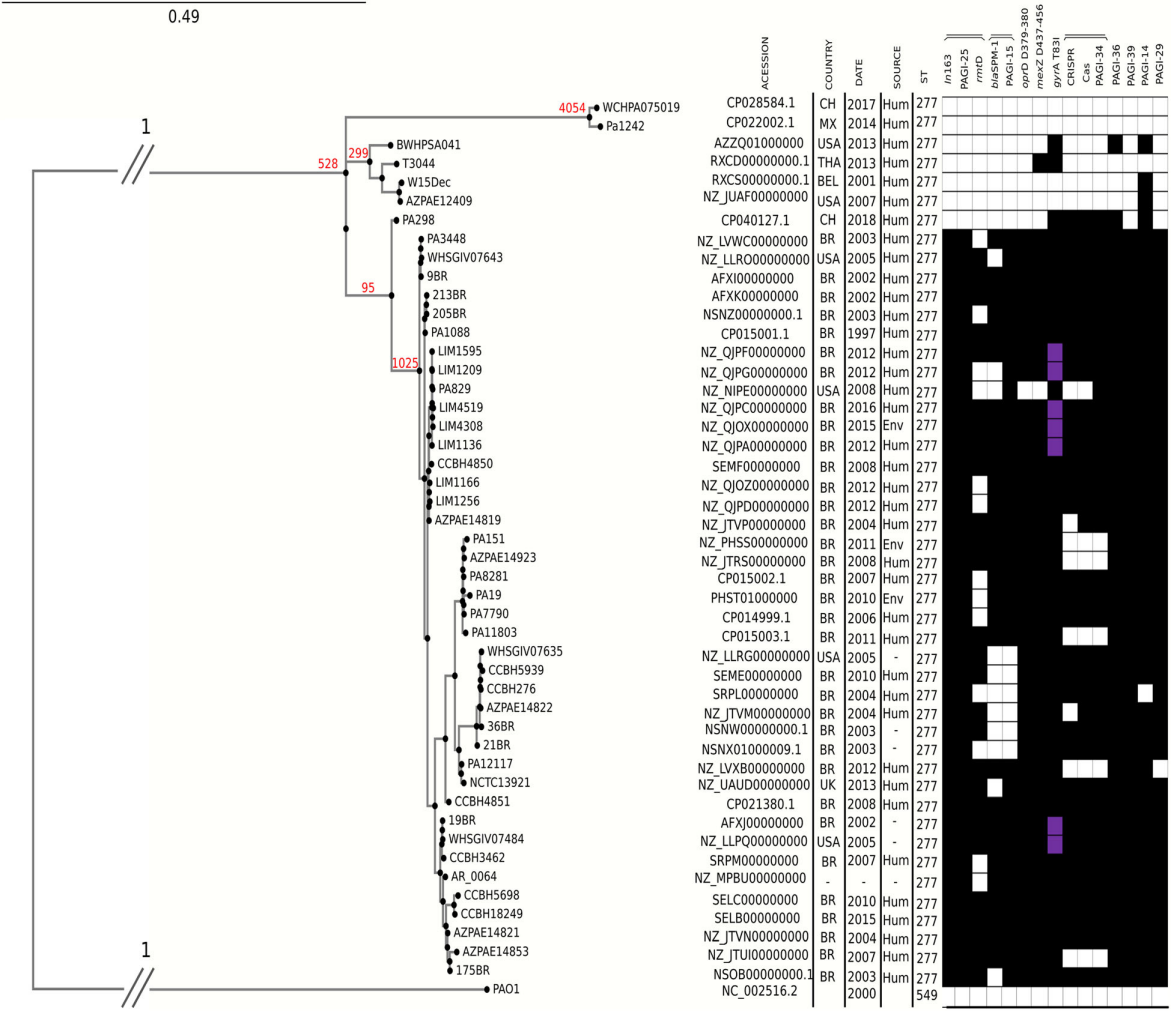
Whole-genome SNP-based parsimony tree of 47 ST277 *P. aeruginosa* isolates and the reference genome PAO1 generated by kSNP3.0. The branch lengths are expressed in terms of changes per number of SNPs. The tree was visualized using Dendroscope. Labels in the internal nodes (red) are the number of SNPs that are exclusively shared by the descendants of that node. The panel shows the presence (black) and absence (white) of the genetic determinants surveyed. The purple bars represent an additional mutation in the *gyr*A gene (D87N). BR: Brazil, CH: China, MX: Mexico, USA: United Stated of America, UK: United Kingdom, BEL: Belgium, THA: Thailand, Hum: Human, Env: Environmental, −: not available

We compared the genome sequence of 47 strains to understand the genomic diversity of *P. aeruginosa* ST277. According to NCBI’s BioSample records, strains from a 21-year period were included in this study (1997–2018). The vast majority was obtained from Brazil (35/47), and overall, they represent human-derived isolates (32/47). The other countries represented are United States (6), China (2), United Kingdom (1), Mexico (1), Thailand (1), and Belgium (1). Based on phylogenetic analysis and SNP differences, we can divide the strains into four important groups. One group with strains from China and Mexico (sharing 4054 exclusively SNPs); another one with strains from the United States, Thailand, and Belgium (sharing 299 exclusively SNPs); a main clade that includes all Brazilian strains plus four strains from the US and one from UK (sharing 1025 exclusively SNPs); and finally a branch containing the Chinese strain (PA298) that share 95 exclusively SNPs with the main clade. Overall, the genomes’ phylogenetic relationships do not seem to be associated to the year of isolation (Fig. 1).

We searched in silico for resistance genes in all ST277 strains. These data demonstrate that 83% (39/47) of the genomes are positive for *In*163, 62% (29/47) for *bla*_SPM-1_ (carbapenem resistance), 57% (27/47) for *rmtD* (aminoglycoside resistance), and 53% (25/47) for *crpP* (ciprofloxacin resistance) and *aac (6′)-IIc* (aminoglycoside resistance). All ST277 strains carry the genes *bla*_OXA-50_ (beta-lactam resistance), *bla*_PDC-5_ (beta-lactam resistance, *Pseudomonas*-derived cephalosporinases), *catB7* (chloramphenicol resistance), *bcr1* (bicyclomycin resistance), *fosA* (fosfomycin resistance) and *aph* (3′)-IIb (aminoglycoside resistance). The prevalence of *bla*_OXA-50_, *bla*_PDC-5_, *catB7*, *bcr1*, *fosA* and *aph (3′)-IIb*, among 2576 *P. aeruginosa* whole-genome shotgun assemblies available at NCBI, are higher than 97% [16]. Therefore, these genes are related to the *P. aeruginosa* species, and not exclusively to the ST277 (Fig. 1, Additional file 1).

Non-synonymous mutations were found in the following resistance related genes: *mexT*, *oprD*, *mexZ*, *nalC*, *pmrA*, *gyrA* and *phoQ*. All strains presented an 8 nucleotide deletion on *mexT* (240–247), besides 39 strains (83%) with 2 nucleotide deletion on *oprD* (379–380) and 19 bp deletion on *mexZ* (437–456), which caused a frameshift. All ST277 strains have the same amino acid alterations in OprD (T103S, K115T), NalC (S209R, G71E), and PmrA (L71R). Forty-two strains have the T83I mutation on GyrA in addition to a D87N mutation on the same protein observed for 7 strains (1.5%), and one strain has a mutation on PhoQ (V260G) (Fig. 1, Additional file 1).

Mutations in the virulence genes were analyzed using the genes from *P. aeruginosa* PAO1 as a reference. Seventeen strains (36%) have different point mutations or one nucleotide deletion in the *lasR* gene, 5 strains (11%) have the same non-synonymous mutation on *rhlR* (K222T), and a fragment with 11 nucleotides was absent in *algB* (382–393) for 3 strains (6%). All ST277 strains carry the virulence genes *exoS*, *exoT* and *exoY* (Fig. 1, Additional file 1).

Genomic comparisons using the pangenome as a reference visually showed that some regions are not shared by all ST277 genomes (Additional file 2). We verified that these regions were PAGIs, already described in previous works [8, 17], or a 49 Kb plasmid, present in 4 strains (36BR, 9BR, PA7790 and PA3448). Samples from countries other than Brazil markedly show the absence of islands such as PAGI-14, PAGI-15, PAGI-25, PAGI-29, PAGI-34, PAGI-36 and PAGI-39 (Fig. 1). Some of these PAGI harbor typical ST277 genetic determinants. For all strains positive for the *bla*_SPM-1_ gene, it is inserted inside PAGI-15. For those strains that carry the class 1 integron *In*163 and/or *rmtD* gene, they are contained in PAGI-25.

In silico analysis identified intact type I-C CRISPR-Cas systems in 33 genomes from ST277 (70%) (Fig. 1, Additional file 1). Two *P. aeruginosa* isolates (AZPAE14819 and AZPAE14822) have the complete Cas operon, but the CRISPR associated was not detected. No other type of CRISPR-Cas system was found among the ST277 strains. When present, the type I-C CRISPR-Cas system was uniformly localized on PAGI-34 in the ST277 genomes analyzed here.

**Fig. 2:**
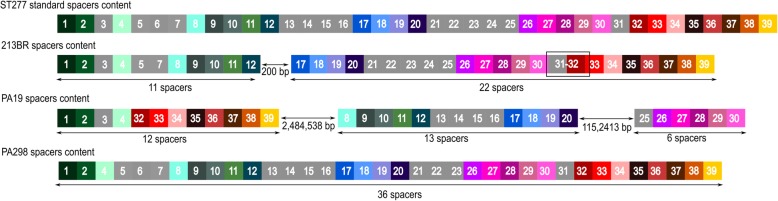
Spacer content among ST277 *P. aeruginosa* carrying type I-C CRISPR-Cas system. Light gray spacers are those not shared between all strains

We inspected the whole spacers content present in type I-C CRISPR-Cas system identified in *P. aeruginosa* genomes. Among the ST277 strains with intact type I-C CRISPR-Cas systems, 91% (30/33) have the same 39 spacers content, considered by us the standard spacers content of this ST (Additional file 3). This data suggests a strong link between clonality (ST) and spacer content. Strain 213BR has a CRISPR array of 22 and another of 11 spacers, separated by 200 bp from each other and near the Cas operon. These 33 spacers are included in the ST277 standard spacers content. We detected 3 CRISPR arrays in the strain PA19, with 12, 13 and 6 spacers, all of them included in the ST277 standard spacers content. The first CRISPR array (12 spacers) is associated with the Cas operon. Strain PA298 has a set of 36 spacers, associated with the Cas operon and included in the ST277 standard spacers content. We noticed that 64% (25/39) of the spacers are present in all genomes from this ST. We need to consider that possible sequencing and assembly errors may have influenced the spacers identification on 213BR and PA19 partially assembled genomes, so the percentage can be higher than 64%. The ST277 spacers at both ends are conserved between the strains, and the deleted/unidentified spacers are positioned in CRISPR central region (Fig. 2).

**Fig. 3:**
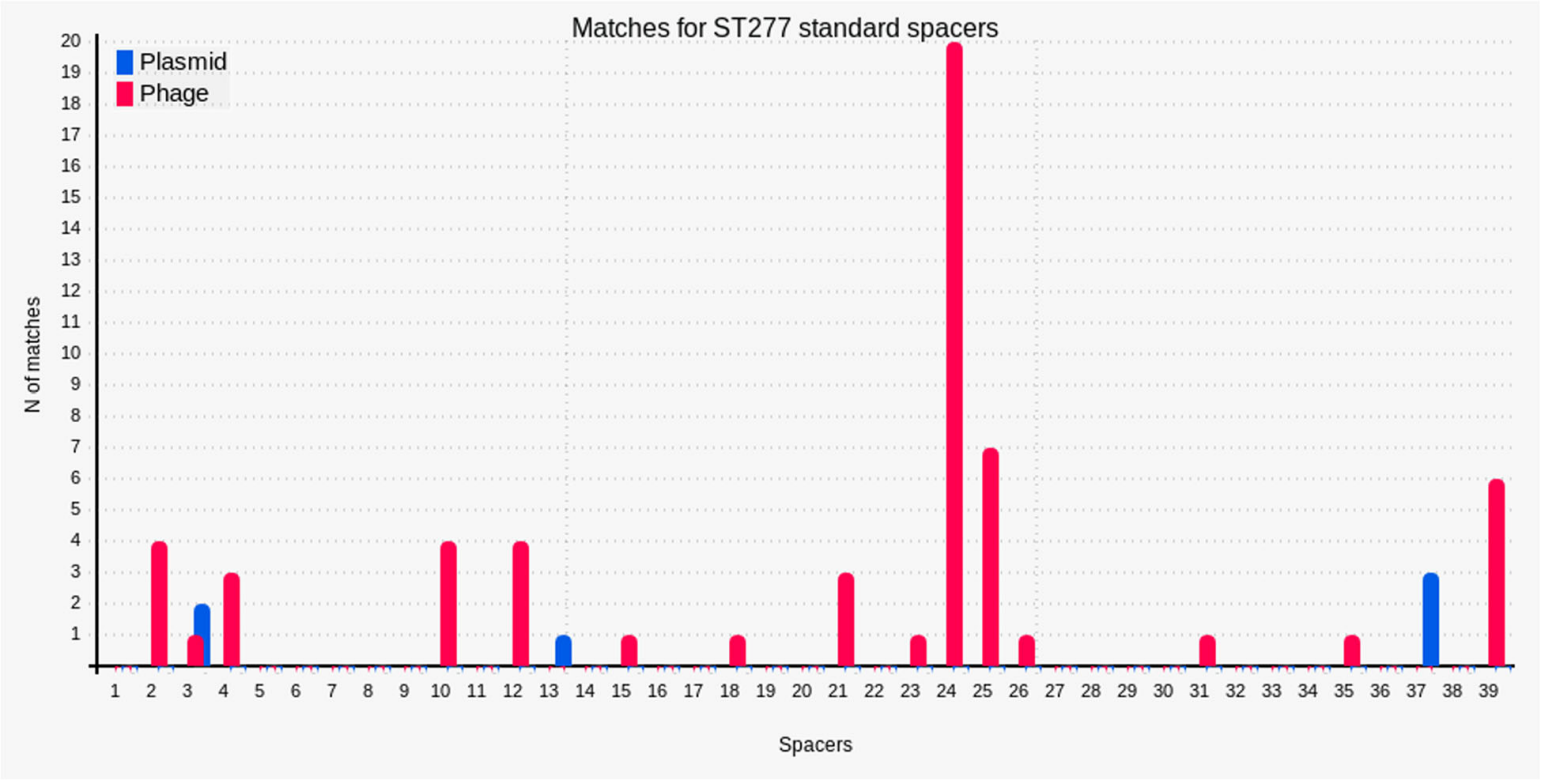
Number of ST277 spacers’ matches against public sequences of plasmids and phages. N: Number of matches

Analyzing BLAT results between the ST277 standard spacers content and the NCBI plasmid and phage databases, targets were assigned for 8% (3/39) and 38% (15/39) of the unique spacers, respectively. In some cases, a unique spacer presented homology with more than one phage and plasmid. We observed that the spacers with the greatest number of matches are distributed throughout the CRISPR array (Fig. 3, Additional file 4).

Among the available plasmid and phage in Genbank, 6 plasmids and 40 phages were targeted by some spacers (Additional file 4). Two target plasmids harbor an integron encoding resistance to aminoglycosides, beta-lactam, and sulfonamides (pPB354_1 and pPB353_1). Among the phages, 15 perform a lysogenic cycle, 2 a lytic cycle, 2 lytic-lysogenic switch, and for 21 we could not find this information using the Genbank accession numbers.

We searched for anti-CRISPR genes (*acr*) on phage and plasmids targeted by CRISPR spacers. Proteins were identified as candidate anti-CRISPRs in 3 plasmids (3/6) and 20 phages (20/40) (Additional file 5, 6 and 7, respectively). The 2 plasmids harboring resistance genes described above also code for putative Acr proteins. Between the 23 anti-CRISPRs candidate genes found in this study, 67% were identified as known groups of *acr* genes, all of them characterized as anti-type I-F (Additional file 4).

## Discussion

*P. aeruginosa* ST277 is a clone spread throughout the Brazilian territory and related to high mortality rates [2, 10, 18]. A greater similarity within Brazilian strains was found when compared to ST277 genomes isolated from other countries, showing that strains from Brazil share a high number of exclusively SNPs.

It has been reported that *In*163, *rmtD* and *bla*_SPM-1_ are frequently associated to ST277 [2, 10, 17, 18]. Together, these mechanisms cause resistance to the main antimicrobials used against *P. aeruginosa* in clinical practice. *In*163 carries 3 gene cassettes that confer resistance to aminoglycosides and cephalosporins [10], and in this work, it was absent only in strains from countries other than Brazil, proving to be a relevant genetic acquisition of ST277 circulating in this territory. The rRNA methylase RmtD promotes a high-level resistance to all clinically available aminoglycosides and was observed in more than half of the ST277 strains studied [11]. SPM-1 producing *P. aeruginosa* is almost exclusively reported in Brazil, mostly belonging to ST277 [2]. Analyzing genomes available from Genbank, we identified that *bla*_SPM-1_-negative ST277 strains are mainly from countries other than Brazil (61%). Only one non-Brazilian sample was positive for *bla*_SPM-1_ (WH-SGI-V-07484, from USA), and more sample information is needed to discuss this observation.

According to our findings, the arsenal of acquired antibiotic resistant genes characteristic of ST277 seems relatively restricted (3 β-lactamase families and 4 aminoglycoside resistant genes) and strains from this clone were mostly isolated in Brazil (74%). This scenario differs from other *P. aeruginosa* high-risk clones such as ST235, which shows a worldwide distribution (more than 20 countries) and possesses a great diversity of resistant genes including 9 β-lactamase families and 22 aminoglycoside transferases genes [19].

We analyzed mutational events in some genes that can contribute to enhance the bacterial resistance phenotype. Among these genetic alterations, those already described as related to increased resistance are: activator deletion of *mexT* that leads to low sensitivity to chlorophenylcholine and fluoroquinolones [20]; *mexZ* nucleotide deletion associated to aminoglycoside, cefepime, tetracyclines and macrolides resistance [8, 21]; mutation in PhoQ linked to colistin-resistant phenotype [22]; GyrA alterations creating quinolone-resistant strains [23]; and *oprD* frameshift related to imipenem resistance [8, 24]. On the other hand, the literature does not associate the PmrA and NalC amino acid changes observed here to resistant strains [25, 26]. In addition, the 20 *oprD* mutations identified for all strains may be a feature of ST277, as specific amino acid substitutions in OprD have been potentially associated to MLST profiles [24].

Changes in genes encoding virulence-related proteins have shown the possibility of increased virulence such as, for example, by the efflux of molecules from the quorum sense signaling system through MexEF-OprN (caused by *mexT* deletion in all strains analyzed) [20]. Some strains presented alteration on *lasR* and *rhlR* that can affect virulence and a wide range of metabolic functions by quorum sensing (QS) regulation [27]. However, other factors found may decrease the virulence of strains, such as the deletion on *algB* in a few strains, that can decrease strain virulence as AlgB is related to the mucoid phenotype [28] and e*xoS* positivity for all strains, which is correlated with a less virulent profile than *exoU* strains [29].

The presence of the type I-C CRISPR-Cas system was first reported in *P. aeruginosa* just recently, mainly associated to STs 277 and 235. This system is located at PAGI-34, a pKLC102-like ICE, the first putative mobile genetic element with a CRISPR-Cas system in a *Pseudomonas* species [9]. Our results confirmed the high prevalence of this subtype on ST277 (70%).

Being aware of the evolutionary and immunological information provided by CRISPR spacers [14], we analyzed the whole spacers content present in type I-C CRISPR-Cas subtype identified in *P. aeruginosa* genomes. We observed a standard spacers content for ST277, and this strong link between sequence types and spacer content in *P. aeruginosa* clinical isolates was already reported previously [9]. This set varied a little for only 3 strains. We believe this may be associated with problems in the sequencing and/or assembly steps of the genomes deposited at NCBI. The exception is PA298, an isolate from China of which the lack of 3 spacers may be related to geographical distance.

We also analyzed the phages and plasmids that were the possible spacers’ targets. The rates of spacers with assigned targets were close to those found by Belkum and coworkers, who used the same identification strategy [9]. Most spacers with matches originated from phage sequences (38%), which are in accordance with the origin of the CRISPR-Cas system, primarily engaged in antivirus defense [30].

A trend related to the presence of a lower fraction of spacers with matches going from the beginning to the end of CRISPR arrays has been described [30]. We found an irregular distribution of the spacers with the greatest number of matches throughout the CRISPR array. As we are studying a specific Cas subtype in a particular *P. aeruginosa* clone this may be influencing the uncommon distribution found here.

Strains with intact CRISPR-Cas systems can be phenotypically CRISPR-Cas incompetent if they encode a cognate anti-CRISPR protein (Acr) that deactivates the corresponding CRISPR-Cas system [9]. The anti-CRISPR-associated protein 1 (Aca1) is a highly conserved sequence located downstream of the *acr* gene, the latter with variable sequences classified in different families [31, 32].

We did not find homologues to the unique anti-type I-C gene described until now (*acr*IC1 from *Moraxella bovoculi*) [33]. However, the dual specificity of some anti-CRISPR proteins has already been described. AcrF6Pae can inactivate both type I-F and I-E CRISPR-Cas systems [31], while AcrVA3.1 may inhibit the type I-C as well as type V-A system [33]. So, all putative Acr proteins identified in this work, even those with anti-type I-F proven activity, should be tested for activity in strains of *P. aeruginosa* possessing the type I-C system.

## Conclusion

The published literature on ST277 *P. aeruginosa* points to a great similarity between these strains, but these studies are always restricted to the Brazilian strains, mostly SPM-1 producers [2, 8, 34]. The hypothesis that ST277 carries an “intrinsic resistome” has been speculated [34]. However, our findings suggest that the proposed “chromosomal pack of resistance genes” is a characteristic of the ST277 strains harboring *In*163 and PAGI-25, circulating mainly in Brazil and accumulating other resistance determinants. More information is required about the USA and UK registered strains that have the same profile to establish a possible correlation between them and the Brazilian strains.

It was still impossible to confirm if this ST appeared as a high-risk clone in this country or if it had already arrived in Brazil with important mechanisms of resistance. The strain collection explored here is of limited size and suffers from a bias in geographical distribution, with a high representation of Brazilian strains. However, this is the most comprehensive genomic analysis of ST277 possible considering the genomes available at the time.

## Methods

### Selection of ST277 *P. aeruginosa* isolates

Seven clinical *P. aeruginosa* ST277 isolates deposited in the Culture Collection of Hospital-Acquired Bacteria (CCBH) located at the Laboratório de Pesquisa em Infecção Hospitalar Hospital/Fiocruz (LAPIH-IOC) (WDCM947; CGEN022/2010) were sequenced on an Illumina MiSeq instrument (Illumina Inc., San Diego, CA, United States) at the Genomic Lab located in the Departamento de Bioquimica, UERJ (Rio de Janeiro, Brazil). The reads were de novo assembled using A5 assembly pipeline [35]. Four of these samples are part of a previous study (CCBH276, CCBH3462, CCBH4851 and CCBH5939) [17]. Posteriorly, CCBH4851 was fully assembled (to be published) (CP021380.1).

Until September 2019, there were 4910 *P. aeruginosa* assembled genomes deposited in NCBI. To identify the genomes belonging to ST277, we picked up the exact matches resulting from BLAST (blastn) [36] searches with the seven alleles for ST277 (retrieved from the MLST databases, https://pubmlst.org/paeruginosa/). By this analysis, 47 genomes were assigned to ST277 (0.95%) and included in this study.

### ST277 phylogeny and Pangenome analysis

For not-completely closed *P. aeruginosa* ST277 genomes, the contigs were reordered using the “reorder contigs” option in Mauve, under the default parameters [37]. The strain *P. aeruginosa* CCBH4851 (CP021380.1), deposited in CCBH-IOC, was used as the reference genome [38].

Phylogenetic analysis of the 47 ST277 genomes and the *P. aeruginosa* PAO1 (NC_002516.2) was performed using kSNP3.0 software [39]. The program Kchooser, which is part of the kSNP package, was used to identify the optimal kmer length (21). Dendroscope was used for visualizing and rooting the parsimony tree by using *P. aeruginosa* PAO1 [40].

The GView Server was used to obtain the pangenome of the *P. aeruginosa* PAO1 and the 47 ST277 isolates with a minimum identity of 90% and an e < 10–5 [41]. The strain CCBH4851 was used as seed genome. Then, genome comparisons were performed with the BLAST Ring Image Generator (BRIG, 0.95) using the pangenome as a reference [42]. The ST277 PAGIs described in previous works were labeled in this comparison using the coordinates obtained by BLAST (blastn) [36] against the pangenome [8, 17].

### Screening of resistance and virulence genes in *P. aeruginosa* ST277 genomes

The prediction of the resistome was made using the Comprehensive Antibiotic Resistance database (CARD) [16]. *In*163 was verified by BLASTn [36] searches using GenBank accession AY660529.1. Besides that, BLAST (blastn) alignment results using PAO1 genes as query were analyzed to identify mutations in some genes related to resistance (not included in CARD analyzes) and virulence. Genes were obtained from *Pseudomonas* genome database (http://www.pseudomonas.com) [*oprD* (PA0958), *mexT* (PA2492), *mexZ* (PA2020) *lasR* (PA1430), *rhlR* (PA3477), and *algB* (PA5483)]. Moreover, BLAST (blastn) was applied to check the presence of the virulence genes *exoS* (AY029246.1), *exoU* (KX641457.1), *exoT* (PA0044, PAO1), and *exoY* (PA2191, PAO1).

### CRISPR-Cas and anti-CRISPR annotation in *P. aeruginosa* ST277 genomes

From reordered contigs or complete genomes, CRISPR arrays and their associated proteins (Cas) were predicted using CRISPRCasFinder [43]. The .json file output for each genome was downloaded and used to extract the CRISPR spacers applying in-house Perl scripts. These scripts considered the orientation of the direct repeat and CRISPR to determine the correct orientation of the corresponding spacer sequences. All the CRISPR arrays with more than two spacers had their spacers clustered together using CD-HIT [44] at a 90% identity threshold to obtain a non-redundant spacer set.

Putative anti-CRISPR (*acr*) genes were identified based on BLAST (blastn) [36] matches to nucleotide sequences of anti-CRISPR-associated protein 1 (Aca1, accession YP_007392343). The proteins encoded by genes immediately upstream of putative Aca1 homologue were selected [31]. Eighteen curated *acr* genes were retrieved from previous studies and aligned to the putative *acr* identified here using MUSCLE software [45] [anti-I-F (*n* = 12), anti-I-E (*n* = 4), anti-I-C (*n* = 1) and anti-VA (*n* = 1)] [31, 33, 46, 47].⁠.

### Target identification for spacer from type I-C CRISPR-Cas system located at *P. aeruginosa* ST277 genomes

When using the non-redundant spacer set as BLAT query (version: 36, parameters: blat -tileSize = 8 -minScore = 28 [reference] [query] [results]) [48], following Belkum et al. 2015 [9], we identified the spacer matches against two categories of sequence databases: 1) phage genomes from NCBI (*n* = 4634 phage files, source: ftp://ftp.ncbi.nlm.nih.gov/genomes/genbank/viral/) (downloaded on April 2018), and 2) Refseq plasmids from NCBI (*n* = 13,428 sequences, source: ftp://ftp.ncbi.nlm.nih.gov/refseq/release/plasmid/ -downloaded on April 2018-).

## Supplementary information

**Additional file 1. **General information about ST277 *P. aeruginosa* NCBI genomes.

**Additional file 2. ***P. aeruginosa* ST277 pangenome.

**Additional file 3. **Spacers from ST277 *P. aeruginosa* CRISPR-Cas system type I-C.

**Additional file 4. **BLAT output from *P. aeruginosa* ST277 spacers against plasmids and phages.

**Additional file 5.** Acr putative sequences.

**Additional file 6.** Acr nucleotide sequences from plasmids.

**Additional file 7.** Acr nucleotide sequences from phages.

## Data Availability

The genomes are available at GenBank - NCBI, and accession numbers are in Fig. 1. Most other relevant data are contained in Additional files. Other datasets used and/or analyzed during the current study are available from the corresponding author on reasonable request.

## Abbreviations

CCBH: Culture Collection of Hospital-Acquired Bacteria
CRISPR: Clustered Regularly Interspaced Short Palindromic Repeat
ICE: Integrative and Conjugative Element
MLST: Multilocus Sequence Typing
PAGI: *Pseudomonas aeruginosa* genomic island
SNP: Single Nucleotide Polymorphism
ST: Sequence type
