# Supplementary figures and images for: Exploring the success of Brazilian endemic clone Pseudomonas aeruginosa ST277 and its association with the CRISPR-Cas system type I-C
Source: BMC Genomics. 2020 Mar 23;21:255. doi: 10.1186/s12864-020-6650-9 (PMC7092672; doi:10.1186/s12864-020-6650-9)

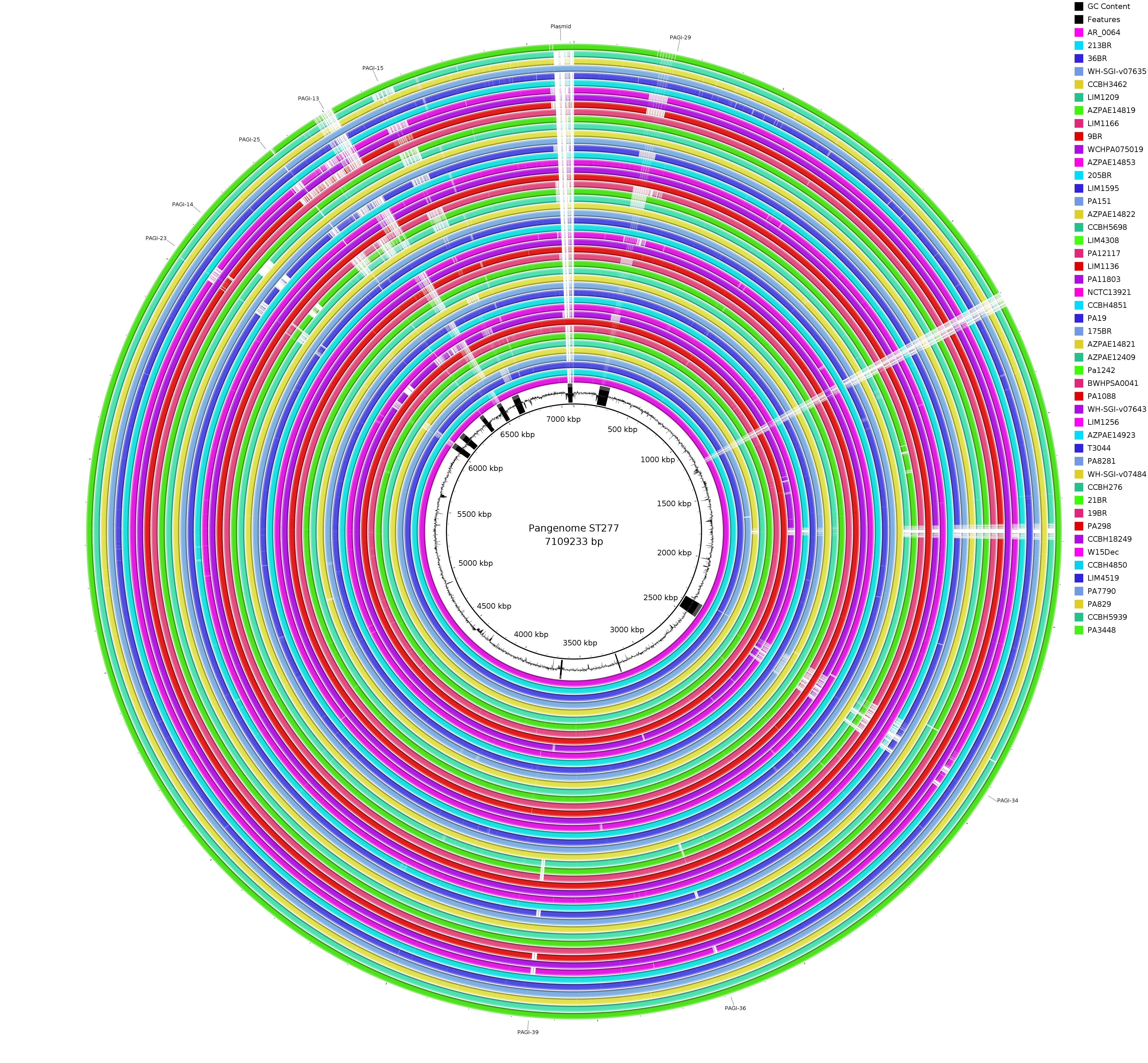

Supplement: Supplementary file 2 — Additional file 2. P. aeruginosa ST277 pangenome. [file 12864_2020_6650_MOESM2_ESM.jpg]
